# Short-term obstetrical and neonatal complications in women with endometriosis

**DOI:** 10.1007/s00404-026-08519-8

**Published:** 2026-07-22

**Authors:** Noa Leybovitz-Haleluya, Gil Gutvirtz, Anastasia Bendet, Eyal Sheiner

**Affiliations:** Department of Obstetrics and Gynecology, Faculty of Health Sciences, Soroka University Medical Center, Ben-Gurion University of the Negev, Beer-Sheva, Israel

**Keywords:** Endometriosis, Cesarean Delivery, Fertility

## Abstract

**Objective:**

Endometriosis is a chronic gynecological condition that affects women in their reproductive age and could potentially lead to reproductive difficulties. Despite extensive research into the impact of endometriosis on fertility and reproductive health, its association with obstetric complications and short-term neonatal outcomes is still an area of limited understanding with conflicting evidence in published studies. We conducted this study to explore a potential effect of endometriosis on obstetric and neonatal outcomes.

**Methods:**

A retrospective population-based cohort study was conducted comparing obstetric and perinatal complications in women with and without endometriosis. We evaluated all singleton deliveries that occurred between 1991 and 2021 at a tertiary medical center. Fetuses with chromosomal abnormalities or congenital malformations were excluded. Generalized estimation equation (GEE) models were used to control for maternal recurrence in the cohort and for confounding variables.

**Results:**

During the study period, 232,476 deliveries met our inclusion criteria, 0.1% (*n* = 224) of whom had documented endometriosis. In the univariate analysis, women with endometriosis were significantly older, had higher rates of infertility and diabetes mellitus, and were more likely to undergo cesarean delivery. They also had higher rates of preterm birth and low birthweight infants, whereas perinatal mortality rates were comparable between the groups. In the GEE models, after controlling for maternal age, gravidity, and fertility treatments, the association for most of the aforementioned variables was no longer statistically significant. However, women with endometriosis were found to have a significant independent higher risk for cesarean delivery (adjusted OR 2.14 (1.62—2.83, *p* < 0.001).

**Conclusion:**

Among the obstetrical and perinatal complications investigated in this study, endometriosis was found to be an independent risk factor for cesarean delivery, while all other pregnancy outcomes were comparable to women without endometriosis.

## What does this study adds to the clinical work

**Table:** 

This study contributes to the existing literature by demonstrating that endometriosis is independently associated only with an increased likelihood of cesarean delivery. These findings may support more evidence-based antenatal counseling and obstetric decision-making, while avoiding unnecessary intervention based solely on the diagnosis of endometriosis.	

## Introduction

Endometriosis is an estrogen-dependent inflammatory disease that affects females during their different hormonal stages, and mainly in the reproductive years. It is estimated that approximately 10 percent of reproductive-age females have endometriosis [1, 2]. However, its prevalence varies significantly in different populations and is difficult to assess as only until recently, definitive diagnosis required invasive investigation.

Common presenting symptoms include chronic or cyclic abdominal and pelvic pain, dysmenorrhea, dyspareunia, and heavy menstrual bleeding [3–5]. It is also considered a major contributor to female infertility [3] and may have a determinantal effect on pregnancy and obstetric outcomes.

During pregnancy, small peritoneal endometriosis lesions may undergo decidualization or regression, and the pain symptoms often improve [6]. However, some case reports have described complications caused by endometriosis in pregnant individuals, including intestinal perforation [7], hemoperitoneum [8, 9], uroperitoneum [10], acute appendicitis [11], and ruptured or infected ovarian endometrioma [12]. Possible mechanisms of endometriosis-induced complications during pregnancy include traction by the growing uterus on adhesions, increased friability of inflamed tissues, and alteration of vessel walls by decidualized lesions [12].

The body of evidence regarding endometriosis and obstetrical complications is controversial. Some studies suggest that endometriosis negatively impacts pregnancy outcomes, although not all studies support this association [13]. Saraswat et al. demonstrated increased risk of miscarriage, ectopic pregnancy, placenta previa, hemorrhage, small for gestational age newborns, and preterm birth among women with endometriosis [14]. In another study, women with untreated rectovaginal deep infiltrating endometriosis had higher rates of pregnancy and delivery complications, such as preterm birth, placenta previa, placental abruption, hypertension, and cesarean delivery, compared with women without endometriosis. Additional adverse pregnancy outcomes including preterm birth, placenta previa, hemorrhage, and low birth weight among were also reported for women with endometriosis [15, 16]. However, other studies found no significant differences in terms of gestational diabetes, hypertensive disorders, fetal growth restriction, intrahepatic cholestasis of pregnancy, placental abruption, or preterm premature rupture of membranes [17].

In this study, we aimed to investigate the association between endometriosis and short-term obstetrical and neonatal outcomes in a large cohort of parturients in a single medical center.

### Study design

This is a population-based retrospective cohort study in which we included singleton deliveries between the years 1991–2021 at Soroka University Medical Center (SUMC), a sole tertiary medical center in the south of Israel. Exclusion criteria were known fetal chromosomal or congenital abnormalities. These exclusion criteria were applied prior to all analyses, and the same eligible cohort was used consistently throughout the study.

Maternal characteristics, demographics, and perinatal complications were compared between women with and without endometriosis. Perinatal complications included diabetes mellitus, hypertensive disorders of pregnancy, gestational age, mode of delivery, preterm delivery, neonatal low birthweight, and perinatal mortality.

This study was based on the computerized perinatal and obstetrical database of the Obstetrics and Gynecology department in SUMC, which is affiliated with CLALIT health maintenance organization (HMO). The database consists of information recorded immediately following delivery, including maternal characteristics and obstetrical, maternal, and neonatal outcomes. In addition, data were validated by medical specialized secretaries in order to make sure the collected data are accurate. Records were anonymized prior to analysis. The study protocol received the approval of the Institutional Review Board Committee of SUMC (0357–19-SOR). Given the retrospective design of the study, based solely on review of existing medical records without direct patient contact or identifiable information, informed consent was not required.

Statistical analysis was performed using the SPSS package 29th ed. Differences in categorical data were assessed by chi-square for general association. T-test was used for comparison of continuous variables with normal distribution. A generalized estimation equation (GEE) binary logistic model was constructed to study the association between endometriosis and different adverse outcomes, while adjusting for maternal recurrence in the cohort and for confounding variables including maternal age, gravidity, and fertility treatments. All analyses were two-sided and a p value < 0.05 was considered statistically significant.

## Results

During the study period, 232,476 deliveries met inclusion criteria, 0.1% (*n* = 224) were of women diagnosed with documented endometriosis. Maternal demographic and clinical characteristics are presented in Table 1.

**Table 1:** Maternal characteristics of women with and without endometriosis: Univariate analysis

	Endometriosis	No Endometriosis	Crude OR	*P* value
	*N* = 224	*N* = 232,252	(95%CI)	
Maternal age (years ± SD)	31.6 ± 5.0	28.1 ± 5.8		< 0.001
Fertility treatments (*N*, %)	97 (43.3)	4791 (2.1)	36.26 (27.80–47.30)	< 0.001
Diabetes mellitus ^a^ (*N*, %)	24 (10.7)	11,501 (5.0)	2.30 (1.51–3.52)	< 0.001
Hypertensive disorders ^b^ (*N*, %)	15 (6.7)	10,760 (4.6)	1.48 (0.88–2.50)	0.139
Smoking	3 (1.3)	1429 (0.6)	2.19 (0.70–6.86)	0.161
Obesity	3 (1.3)	2702 (1.2)	1.15 (0.37–3.61)	0.806

^a^ including pre-gestational and gestational diabetes mellitus

^b^ including pre-gestational hypertension, gestational hypertension, and preeclampsia

Women with endometriosis were significantly older (31.6 ± 5.0 vs. 28.0 ± 5.8, *p* < 0.001). These women also had higher rates of infertility (43.3% vs. 2.1%, *p* < 0.001, crude OR 9.82 (5.39—17.86)). In the univariate analysis, diabetes mellitus rates were higher among women with endometriosis (10.7% vs. 5.0%, crude OR 2.30, 95% CI 1.51–3.52, *p* < 0.001). However, this association was no longer significant after adjustment for maternal age, gravidity, and fertility treatments (adjusted OR 1.02, 95% CI 0.64–1.62, *p* = 0.930). No significant difference was found in the rates of hypertensive disorders, smoking status, and obesity among groups.

Obstetrical and neonatal outcomes of both groups are presented in Table 2. Women with endometriosis were more prone to undergo a cesarean delivery, a result that remained significant also after controlling for confounders in a GEE model (46.0% vs. 14.0%, *p* < 0.001, adjusted OR 2.14 (1.62—2.83)).

**Table 2:** Obstetrical and perinatal outcomes of women with and without endometriosis: Univariate analysis

	Endometriosis	No Endometriosis	Crude OR	*P* value
	*N* = 224	*N* = 232,252	(95%CI)	
Preterm delivery (< 37 weeks) (*N*, %)	28 (12.5)	16,342 (7.0)	1.89 (1.27—2.80)	0.001
Cesarean delivery (*N*, %)	103 (46.0)	32,506 (14.0)	5.23 (4.02–6.80)	< 0.001
Low birthweight (< 2500 g) (*N*, %)	25 (11.2)	16,721 (7.2)	1.62 (1.07 – 2.46)	0.023
Perinatal mortality (*N*, %)	3 (1.3)	1883 (0.8)	1.66 (0.53 – 5.19)	0.435

All estimates presented in tables are derived from univariate (crude) analyses. Adjusted estimates from GEE models are reported in the Results section

In the univariate analysis, women with endometriosis had higher rates of preterm delivery (12.5% vs. 7.0%, crude OR 1.89, 95% CI 1.27–2.80, *p* = 0.001) and low birthweight infants (11.2% vs. 7.2%, crude OR 1.62, 95% CI 1.07–2.46, *p* = 0.023). However, In the GEE models, after controlling for maternal age, gravidity, and fertility treatments, the association between endometriosis and preterm delivery (*p* = 0.086, adjusted OR 1.44 (0.95—2.17)) as well as low birthweight (*p* = 0.366, adjusted OR 1.22 (0.79—1.86)) was no longer statistically significant. In addition, in the univariate analysis, perinatal mortality rates were comparable between the groups (1.3% vs. 0.8%, crude OR 1.66, 95% CI 0.53–5.19, *p* = 0.435). This association remained non-significant after adjustment in the GEE model (adjusted OR 1.45, 95% CI 0.47–4.47, *p* = 0.516)*.*

## Discussion

The main finding of this large population-based study is that endometriosis is an independent risk factor for cesarean delivery (CD). Other obstetrical and neonatal adverse outcome were not found to be independently associated with endometriosis. These findings are consistent with prior studies reporting higher rates of CDs among women with endometriosis [16, 18]. However, regarding other obstetrical and neonatal complications, the data from previous studies are controversial. Some studies demonstrated higher rates of placental complications and preterm delivery [19, 20], while other studies have not shown increased risks for obstetric or neonatal adverse outcomes [21]. Mor et al. demonstrated recently in their retrospective study a significantly higher prevalence of inflammatory placental lesions in women with endometriosis compared with controls. These included both acute and chronic placental inflammatory changes, such as chronic villitis, deciduitis, and increased placental hemorrhage [22]. Chronic inflammation associated with endometriosis can also lead to fibrosis and pelvic adhesions, which can potentially distort normal pelvic anatomy and reduce uterine or pelvic organ mobility predisposing to labor dystocia or fetal malpresentation [23], thus increasing the likelihood of CD.

In addition, functional uterine changes have been also proposed. Structural and functional alterations of the myometrium have been documented in women with endometriosis and adenomyosis, including disrupted myometrial architecture, increased fibrosis, and abnormal myometrial contractility, all of which may reduce the efficiency and coordination of uterine contractions during labor [24, 25], which could compromise labor progress and lead to higher rates of CD.

Furthermore, we assume that clinical decision-making and obstetric management also may play a role. Women with endometriosis often have a history of infertility, pelvic pain, or previous pelvic surgery, which may lead obstetricians and patients to favor cesarean delivery as a more controlled and expected mode of delivery. This assumption has to be further studied.

The rate of CD has risen sharply in the last decades worldwide [26]. CD increases the risk for maternal morbidity such as bleeding and infections [27, 28]. CD has also been associated with increased morbidity of the offspring in the long-term in previous studies [29, 30]. We assume also that the physician may be biased when managing labor of these patients, and may be less tolerant to slower labor partograms in women with endometriosis. Therefore, reconsideration by the clinicians of the indications for CD in women with endometriosis is warranted in order to lessen the rising rate of CDs.

The main strength of our study is its large cohort size. In addition, SUMC is the sole tertiary hospital in the entire Negev region in an area characterized by positive immigration, resulting in probable minimal selection bias. However, this study has several limitations that should be acknowledged. The main limitation lies within its retrospective design. As a population-level analysis, our study can provide evidence only of association and not of causation. Nevertheless, there seems to be a biological plausibility for the observed association. In addition, we lacked information on endometriosis severity and method of diagnosis (clinical, imaging-based, or surgical confirmation). Thus, our ability to determine whether specific subtypes of endometriosis carry higher obstetric risk or assess the correlation between disease severity and rate of complications is limited. Also, the timing of endometriosis diagnosis was not available in our database. Therefore, we could not distinguish between women with preexisting endometriosis and those in whom the diagnosis may have been established incidentally at the time of cesarean delivery. Consequently, the possibility of diagnostic ascertainment bias and circular causation cannot be excluded and should be considered when interpreting the observed association between endometriosis and cesarean delivery.

In the final analysis, fertility treatments were analyzed as a binary variable. Therefore, we were unable to differentiate between specific treatment modalities, such as more conservative treatments including ovulation induction or in vitro fertilization techniques, which may have different obstetric risk profiles and associations with endometriosis. In addition, data on the specific clinical indications for CD were unavailable, preventing us from distinguishing between biological risk, labor-related complications, and provider or patient-driven decision-making. Lastly, the prevalence of documented endometriosis in our cohort was substantially lower than that reported in the literature. This finding may reflect under-diagnosis and underreporting inherent to the use of routinely collected clinical data over a 30-year study period, during which awareness and diagnostic approaches to endometriosis have changed considerably. Consequently, some women with endometriosis may have been misclassified as unaffected, potentially attenuating the observed associations.

In conclusion, documented endometriosis was independently associated with an increased likelihood of cesarean delivery, while other obstetrical and neonatal outcomes were comparable to those of women without endometriosis. Given the inability to determine the timing of endometriosis diagnosis, these findings should be interpreted cautiously. Future prospective research is needed to clarify whether the increased cesarean rates reflect true obstetric risk or differences in clinical practice.

## Data Availability

Data are available upon request.
